# Gender Difference in the Effects of Outdoor Air Pollution on Cognitive Function Among Elderly in Korea

**DOI:** 10.3389/fpubh.2019.00375

**Published:** 2019-12-10

**Authors:** Hyunmin Kim, Juhwan Noh, Young Noh, Sung Soo Oh, Sang-Baek Koh, Changsoo Kim

**Affiliations:** ^1^Department of Preventive Medicine, Yonsei University College of Medicine, Seoul, South Korea; ^2^Division of Health Systems Management and Policy, University of Memphis School of Public Health, Memphis, TN, United States; ^3^Institute of Human Complexity and Systems Science, Yonsei University, Incheon, South Korea; ^4^Department of Neurology, Gachon University Gil Medical Center, Incheon, South Korea; ^5^Department of Occupational and Environmental Medicine, Wonju Severance Christian Hospital, Wonju College of Medicine, Yonsei University, Wonju, South Korea; ^6^Department of Preventive Medicine, Wonju Severance Christian Hospital, Wonju College of Medicine, Yonsei University, Wonju, South Korea

**Keywords:** air pollution, gender difference, cognitive function, particulate matter, nitrogen oxide, mini-mental state examination

## Abstract

**Background/Aim:** Given a fast-growing aging population in South Korea, the prevalence of cognitive impairment in elderly is increasing. Despite growing evidence of air pollution exposure as one of the risk factors for declining cognition, few studies have been conducted on gender difference in the relation of cognitive function associated with outdoor air pollution. The aim of this study is to investigate the effect modification of gender difference in the association between cognitive function and air pollutant exposure (PM_10_, PM_2.5−10_, and NO_2_).

**Methods:** The study focused on elderly, and the resulting sample included 1,484 participants aged 55 and older with no neurologic diseases, recruited from the four regions in Korea (Seoul, Incheon, Pyeongchang, and Wonju). We used the Mini-Mental State Examination (MMSE) score (with the conventional cut-off point “23–24”) to assess cognitive decline as the primary outcome of the study. Air pollution data used in this study were based on the 5-year average of predicted PM_10_ and NO_2_ concentrations, as well as the 2015 average PM_2.5_ concentration. Additionally, a survey questionnaire was utilized to obtain information about general health assessment. To explore gender differences in the effects of air pollution exposure on cognitive function, we used penalized logistic regression, negative binomial regression, and generalized linear mixed model analyses. Subgroup analyses were also performed by the geographic location of residence (metropolitan vs. non-metropolitan).

**Results:** We found that women than men had a higher risk for decreased cognitive function associated with increased exposure to PM_10_ and PM_2.5−10_, respectively, even after adjustments for confounding factors (OR 1.01 [95%CI 1.00-1.03] in PM_10_; OR 1.03 [95%CI 1.01–1.07] in PM_2.5−10_). After stratification by metropolitan status, we also found that the adverse effect of NO_2_ exposure on cognitive function was higher in women than men [OR 1.02 [95%CI 1.00–1.05] in metropolitan; OR 1.12 [95%CI 1.04–1.20] in non-metropolitan]. Notably, the magnitude of the effect sizes was greater among those in non-metropolitan regions than metropolitan ones.

**Conclusions:** Although our findings suggest that the adverse effects of outdoor air pollution on cognitive function appeared to be higher in women than men, this should be tentatively reflected due to some limitations in our results. While additional research is warranted to confirm or dispute our results, our findings suggest an indication of the need for developing and implementing prevention or interventions with a focus on elderly women with increased risk for air pollution exposure.

## Introduction

The decline of cognitive function is one of the reasons for low independence and likely disability among older adult populations (1, 2). In South Korea, as one of the countries with a fast-growing aging population, the prevalence of cognitive deficits or decline among older adults (aged 65 years and older) had been increasing considerably (3). A nationwide survey conducted by Kim and colleagues (3) estimated the prevalence of mild cognitive impairment and dementia in elderly people aged 65 and older in South Korea and projected that the expected number of dementia cases would be doubled every two decades before the coming 2050 (3). The burden associated with increased cognitive decline among the elderly is likely to grow with rapidly growing aging populations.

Essential is to reduce risk factors for cognitive decline or impairment. For example, the known risk factors such as age, sex/gender, socioeconomic conditions, genetic component, health risk behaviors (i.e., smoking and drinking), high blood pressure, and disease morbidities were examined in prior studies (4–10). Among risk factors, comparatively less is known about environmental factors, including air pollution, which could have played a role in risk for cognitive decline or deficits (2, 11, 12). In South Korea, in recent years, the issue of ambient particulate air pollution has become more severe than before. This aspect could play a role in adversely affecting the health of the population. Evidence showed that high levels of exposure to PM_2.5_, PM_10_, and NO_2_ were adversely associated with respiratory health (13–16), cardiovascular diseases (14, 17), and increased risk of psychiatric disorders (18).

It has been gradually suggested that there is a relationship between air pollution exposure and cognition in elderly (2, 19, 20). For instance, a prospective cohort study by Weuve et al. (19) examined whether long-term PM exposure may be related to cognitive decline among the US women aged 70–81 years in the Nurses' Health Study Cognitive Cohort and found that higher levels of PM_2.5−10_ and PM_10_ exposure were related to faster decline in cognitive function, respectively. Meanwhile, Ailshire and Crimmins (20) explored the relation of cognitive function associated with high concentrations of PM_2.5_ among older US adults using data from the Health and Retirement Study. They found that high levels of PM_2.5_ exposure had an adverse effect on cognitive function, but no notable significant effect modification by individual-level characteristics, including demographics and socioeconomic factors was found.

In spite of growing indication of a positive link between air pollution exposure and cognitive decline, much less consideration has been directed to the extent of gender difference in the relation of cognitive function associated with air pollution exposure. Although a few studies explored effect modification by gender/sex as a supplementary purpose concerning examining the main effects of air pollution on cognitive function (11, 20), the results were varied. Specifically, previous research found that the effect of air pollution exposure on cognition was different between males and females (21, 22), while no such result was found for other research (23). Notably, there is a discrepancy over whether the effect is higher in males than females or vice versa (21–23). Therefore, this study aims to clarify this inconsistency by investigating gender differences in the association between exposure to air pollutants (PM_10_, PM_2.5−10_, and NO_2_) and cognitive functioning in a cohort of Korean older adults. Detecting gender differences and identifying the mechanism can help develop gender-specific interventions or policies to reduce the risk of cognitive decline associated with outdoor air pollution among older adult populations.

## Materials and Methods

### Study Subjects

Participants were included among the cohorts recruited from the four different regions of South Korea (i.e., Seoul, Incheon, Wonju, and Pyeongchang) between 2014 and 2017. Specifically, they were voluntary participants recruited through community advertisement from the Nowon-gu, Yangcheon-gu, Mapo-gu, Seodaemun-gu, Eunpyeong-gu, and Gangnam-gu of Seoul, the Namdong-gu and Ganghwa-gun of Incheon, and Wonju and Pyeongchang, respectively. Since it was voluntary recruitment conducted in small districts, our study has a limitation on generalizability. The inclusion criteria were: aged 55 years or above and no history of having dementia, Parkinson's disease, or stroke. We excluded patients with Alzheimer's disease and amnestic mild cognitive impairment, as well as missing data concerning the characteristics of participants except for smoking status since most of the women were likely to be non-smokers or social smokers. Although we kept them in the study, we did not adjust for smoking status in our regression analyses, which will follow in the statistical analysis section. The resulting sample was comprised of 1,484 participants (569 men and 915 women). We enrolled the participants from Seoul and Incheon, separately, in Gachon University Gil Medical Center and Yonsei University Severance Hospital for a medical examination, and those from Wonju and Pyeongchang in the Lifetime Management Center at Yonsei University Wonju College of Medicine and Wonju Severance Christian Hospital for a medical checkup, respectively. The study was approved by the Institutional Review Boards of the Yonsei University Health System (IRB Approval No. 4-2014-0359). Trained nurses as research staff administered a survey questionnaire, constructed based on the prior studies for outcome assessment (12, 15, 24) as well as the Korea Centers for Disease Control and Prevention for epidemiological characteristics (25), to all subjects following the protocols and ethical standards of the human experimentation committee at Yonsei University Severance Hospital. The study participants were asked to answer the questions about demographics, anthropometric measurement, socioeconomic condition, health risk behaviors (smoking, drinking, etc.), physical activity, disease morbidities including family history, and residential characteristics.

### Outcome Assessment

Mini-Mental State Examination (MMSE) as the most commonly used cognitive test (26, 27) was used to assess participants' cognitive function. Notably, MMSE, which detects individual cognitive impairment by evaluating mental status, is frequently used for dementia screening in the primary care setting (28, 29). MMSE with 30 points in total comprises questions on orientation, attention, calculation, language, and recall (29). The study used the conventional cut-off point for the MMSE (“23–24”) as an indication for a decline in cognitive functioning, based on the criteria employed in the prior studies (12, 30). Therefore, the dependent variable in the study was “decreased cognitive function” as binary (“1” if MMSE score ≤ 23 corresponding to a decreased cognitive function, “0” if 24 ≤ MMSE score ≤ 30 corresponding to a normal cognitive function), and we also included MMSE score as a continuous variable in the models.

### Prediction for Long-Term Concentrations of Particulate Matter and Nitrogen Dioxide

Based on the residential addresses of the participants, each was assigned the predicted exposure concentration based on a national point-wise exposure prediction approach using regulatory monitoring data for 2010. In spite of this prediction, however, we acknowledge some weaknesses in our data: (1) limitation about one chronic exposure incorporated; (2) the presence of a possible regression toward null effect subject to modeled data used without reflecting individual exposure appropriately. However, it should be noted that regression toward null effect is considered as a limitation in most of the studies using modeled air pollutants data. That is, results may be shown with no impact of air pollution due to using the average concentrations of air pollutants, although it may exist when using the actual values. A specific method of prediction was documented in another published article (31).

Specifically, Kim et al. (31) developed the national prediction model by incorporating the annual mean concentration values of log-transformed NO_2_ and PM_10_ estimated at the 277 air quality monitoring sites between 2010 and 2016 after excluding 11 places that did not meet the inclusion criteria. In the process of developing a prediction model, 322 geographic variables that consist of proximity and buffer in eight categories, including traffic, physical geography, land use, demographic characteristics, etc. were used to get information about geographic characteristics that could be attributed to the spatial variability of air pollution. This universal kriging model comprises two elements: mean and variance. That is, the mean was characterized with the two and three predictors which were estimated by partial least squares, while the variance was assessed using the three parameters for covariances such as partial still, range, and nugget which could find the presence of spatial correlation and both spatial and non-spatial variability. Consequently, we calculated the predicted values of the annual average concentrations of NO_2_ and PM_10_ at the residential addresses of the study participants over the past 5 years, using the model developed. The rationale for using the 5-year mean values of the air pollution levels was that the information about participants' residential addresses was solely available in those 5 years. For PM_2.5_ data, however, we merely used the 2015 average concentration as a proxy for the 5-year values because information about PM_2.5_ had become publicly available since 2015.

Concerning air pollution concentration levels in the four regions, Seoul had an average of 22.23 (μg/m3) for PM_10−25_, 47.88 (μg/m3) for PM_10_, and 33.80 (μg/m3) for NO_2_. Incheon had an average of 21.61 (μg/m3) for PM_10−25_, 47.90 (μg/m3) for PM_10_, and 33.33 (μg/m3) for NO_2_. Wonju had an average of 13.86 (μg/m3) for PM_10−25_, 39.37 (μg/m3) for PM_10_, and 10.88 (μg/m3) for NO_2_. Pyeongchang had an average of 16.82 (μg/m3) for PM_10−25_, 42.50 (μg/m3) for PM_10_, and 15.35 (μg/m3) for NO_2_.

This study included “exposure to air pollutants (PM_10_, PM_2.5−10_, and NO_2_)” as continuous variables in the main models, while we also examined them with categorization as part of our sensitivity analysis in the statistical analysis section.

### Potential Confounding Factors

Following the status of the participants, potential confounding factors were included. Age (in years) was included, recoded as a categorical variable (55–64 and 65+), given the fact that older age is a strong risk factor for cognitive decline (7). Other factors such as gender, marital status, education, income, smoking status, drinking, body mass index (BMI), systolic blood pressure, disease morbidities, and geographic location were included, since women, low educational and income levels, being unmarried, smoking and drinking, higher BMI, high blood pressure, and disease morbidities (hypertension, diabetes, hyperlipidemia, and depression) were indicated as risk factors for cognitive decline or impairment in the literature (4, 6, 7, 10, 32–34). Marital status indicating whether an individual is currently married was recoded and included as a dichotomous variable (yes or no). As indicators for socioeconomic condition, education (“less than middle school” and “middle school graduate or more”) and income (X, Korean won) (X <1,000,000, 1,000,000 ≤ X <2,000,000, 2,000,000 ≤ X < 4,000,000, and X ≥ 4,000,000) were included. Smoking status was assessed based on the questions of whether a participant smoked more than five packs of cigarettes in each entire life and if he or she currently smokes (yes or no), which was categorized into “current smoker,” “former smoker,” and “never smoker.” Alcohol drinking was assessed based on the question of whether a participant does not drink at all, which was recoded and included as a binary variable. We calculated BMI (kg/m^2^) using individual weight and height and included it as a categorical variable [underweight (<18.5), normal weight (18.5–22.9), overweight (23.0–24.9), and obese (≥25.0)], based on Asia-Pacific BMI classifications (35, 36). Disease morbidities (hypertension, diabetes, and hyperlipidemia) were included, indicated by the question of whether or not an individual has ever been diagnosed by a physician, which was conducted through a trained nurse's interview. Blood pressure was measured twice, and average of systolic blood pressure was included in the models. Being depressed was included as a dichotomous variable, assessed by the Korean version of the Short Geriatric Depression Scale (SGDS-K) (37) (i.e., depressed = yes if 8 ≤ SGDS-K ≤15; depressed = no if 0 ≤ SGDS-K < 8). Moreover, we included the variable “geographic location” based on the residence area of the participants (Seoul, Incheon, Wonju, and Pyeongchang), categorized into “metropolitan” (Seoul and Incheon) and “non- metropolitan” (Wonju and Pyeongchang). In addition to those risk factors, we included the variables, physical activity [“often (4 days or above),” “a few times (1 to 3 days),”and “never (<1 day)”]and quality of daily life (“good quality,” “neither poor nor good,” “poor”), as related protective factors indicated by prior studies (38, 39), which were assessed based on the questions, “Recently, how many days did you walk at least 10 min?” and “How would you rate the quality of your daily life? (i.e., work, house chores, leisure activities, etc.), respectively.

### Statistical Analysis

The dependent variable in this study was: “decreased cognitive function” as binary (“1” if MMSE score ≤ 23, “0” if 24 ≤ MMSE score ≤ 30). We performed different regression analyses to detect and examine gender differences in the relationship between air pollution exposure and cognitive function in the participants. Because the dependent variable has extra zero observations after categorizing, in which general logistic regression models may not be adequate to use (40), we first conducted penalized logistic regression analyses to examine the odds of declining cognitive function associated with increased exposure to air pollutants (PM_10_, PM_2.5−10_, and NO_2_). Notably, penalized logistic regression analysis can solve problems, including multicollinearity and overfitting, which commonly occur in the use of general logistic regression models (41). Next, we analyzed negative binomial regression models since we also examine the MMSE score as a count variable with over-dispersion in which the general poisson regression model does not fit adequately. Furthermore, we conducted generalized linear mixed model analyses to include a geographic location (metropolitan vs. non-metropolitan) as random effects concerning examining the effects of air pollution exposure on cognitive function in the participants. To detect and investigate the gender differences, we created and employed two different models: interaction models and stratified models. Specifically, we incorporated interaction terms in the regression analyses, based on the results from the Rao-Scott Chi-square test and a two-sample *t*-test for categorical variables and continuous variables, respectively. Moreover, we conducted regression analyses by stratifying gender.

In a sensitivity analysis, we additionally examined exposure to air pollutants (PM_10_, PM_10−25_, and NO_2_) as interval variables using label values at the 25, 50, and 75 percentiles, respectively to see whether and how the estimated effect of air pollution on cognitive function would change at intervals. Specifically, we divided air pollutants such as PM_10_, PM_10−25_, and NO_2_, respectively, into the four classes: (1) PM_10_: Q1 (PM_10_≤43.65), Q2 (43.65<PM_10_≤47.26), Q3 (47.26<PM_10_≤48.67), Q4 (48.67<PM_10_≤55.40); (2) PM_10−25_: Q1 (PM_10_≤17.91), Q2 (17.91<PM_10−25_≤21.08), Q3 (21.08<PM_10−25_≤22.76), Q4 (22.76<PM_10−25_≤28.33); (3) NO_2_: Q1 (NO_2_≤20.44), Q2 (20.44<NO_2_≤32.34), Q3 (32.34<NO_2_≤35.10), Q4 (35.10<NO_2_≤44.38). We also estimated the effect of air pollution exposure on cognition with the log-transformation as commonly used to deal with non-normal or skewed data (42). It is because our air pollutant data were indicated as non-normal from the result of the Shapiro-Wilks normality test (*p* <0.0001). Thus, we wanted to see whether the effects on cognition would alter with the log-transformed air pollutants. Further, we investigated whether there exists any non-linear effect of air pollution exposure on cognition using zero-truncated negative binomial regression models, which can confirm our results. In addition to looking over and exploring air pollution in multiple ways, we examined MMSE score using a different cut point (“24–25”) since after excluding patients with a previous diagnosis such as Alzheimer's disease and amnestic mild cognitive impairment, many participants were resulted in having MMSE scores ≥25 (87.61%). In this regard, we investigated the effect of outdoor air pollution on cognition by incorporating MMSE score values ≤ 25 as an indication of lower cognition.

The significance threshold was set at 0.05. All statistical analyses were conducted using SAS, version 9.4 (NC, USA).

## Results

Table 1 shows the characteristics of the Korean cohort and the difference by gender. The mean exposure levels of PM_10_, PM_2.5−10_, and NO_2_ were significantly higher in women than in men (46.56, 20.66, and 30.11 μg/m^3^ in women vs. 45.09, 19.24, and 25.83 μg/m^3^ in men). Being >65 years old (74.17 vs. 63.83%), married (93.66 vs. 72.79%), with a high education (52.20 vs. 39.56%) and income (14.94 vs. 10.49%) were significantly more common in men than in women. Doing physical activity (94.10 vs. 91.04%) and having a metropolitan area of residence (85.90 vs. 64.50%) were significantly more prevalent in women than in men. Having a good quality of life (92.96 vs. 88.31%) and a BMI > 25.00 (41.12 vs. 37.70%) were significantly more common in men than in women. Smoking and drinking habits were significantly higher in men than in females: 71 vs. 2.85% for smoking status (both current and past); 71.88 vs. 36.50% for alcohol drinking. For disease morbidities, hypertension and hyperlipidemia were significantly more common in women than in men: 36.17 vs. 26.19% for hypertension; 37.05 vs. 24.96% for hyperlipidemia. On the contrary, diabetes was significantly more prevalent in men than in females: 19.68 vs. 14.43%. Having a decreased cognitive function was significantly more common in females than in men: 10.27 vs. 5.62%. Only systolic blood pressure (128.56 vs. 127.64 mmHg), being depressed (12.83 vs. 15.96%), and MMSE score (27.25 vs. 27.21) did not significantly differ between men and women.

**Table 1:** Characteristics of the study participants.

**Characteristics**	**All (*n* = 1,484)**	**Men (*n* = 569)**	**Women (*n* = 915)**	**Statistical test^a/^**	***p*-value**
	**Mean ± SD^b/^**	**Mean ± SD^b/^**	**Mean ± SD^b/^**		
PM_10_ (μg/m^3^)	46.00 ± 3.93	45.09 ± 4.30	46.56 ± 3.57	*t* = −7.15	<0.0001
PM_2.5−10_ (μg/m^3^)	20.11 ± 3.71	19.24 ± 4.04	20.66 ± 3.38	*t* = −7.26	<0.0001
NO_2_ (μg/m^3^)	28.47 ± 10.19	25.83 ± 11.24	30.11 ± 9.11	*t* = −8.03	<0.0001
Systolic blood pressure (mmHg)	127.99 ± 13.49	128.56 ± 12.91	127.64 ± 13.84	*t* = 1.74	0.082
	N (%)	%	%		
Age				χ^2^ = 17.17	<0.0001
55–64	478 (32.21)	25.83	36.17		
65+	1,006 (67.79)	74.17	63.83		
Marital status				χ^2^ = 98.37	<0.0001
Yes	1,198 (80.78)	93.66	72.79		
No	285 (19.22)	6.34	27.21		
Education				χ^2^ = 22.68	<0.0001
Less than middle school	825 (55.59)	47.80	60.44		
Middle school graduate or more	659 (44.41)	52.20	39.56		
Income (X) (won)				χ^2^ = 11.80	0.008
X <1,000,000	382 (25.74)	23.55	27.10		
1,000,000 ≤ X <2,000,000	493 (33.22)	30.23	35.09		
2,000,000 ≤ X <4,000,000	428 (28.84)	31.28	27.32		
X ≥ 4,000,000	181 (12.20)	14.94	10.49		
Physical activity				χ^2^ = 6.15	0.046
Often	1,040 (70.08)	70.13	70.06		
A few times	339 (22.84)	20.91	24.04		
Never	105 (7.08)	8.96	5.90		
Quality of daily life				χ^2^ = 8.85	0.012
Good	1,336 (90.09)	92.96	88.31		
A little	142 (9.57)	6.69	11.36		
Not at all	5 (0.34)	0.35	0.33		
Ever smoker				χ^2^ = 798.01	<0.0001
Current	86 (5.80)	14.06	0.66		
Former	344 (23.18)	56.94	2.19		
Never	4 (0.27)	0.53	0.11		
Not reported ^c/^	1,050 (70.75)	28.47	97.04		
Drinking				χ^2^ = 175.63	<0.0001
Yes	743 (50.07)	71.88	36.50		
No	741 (49.93)	28.12	63.50		
BMI				χ^2^ = 14.21	0.002
<18.50	25 (1.68)	1.76	1.64		
18.50–22.90	437 (29.45)	23.90	32.90		
23.00–24.90	443 (29.85)	33.22	27.76		
≥ 25.00	579 (39.02)	41.12	37.70		
Depressed ^d/^				χ^2^ = 2.72	0.098
Yes	219 (14.76)	12.83	15.96		
No	1,265 (85.24)	87.17	84.04		
Hypertension				χ^2^ = 15.99	<0.0001
Yes	480 (32.35)	26.19	36.17		
No	1,004 (67.65)	73.81	63.83		
Hyperlipidemia				χ^2^ = 23.42	<0.0001
Yes	481 (32.41)	24.96	37.05		
No	1,003 (67.59)	75.04	62.95		
Diabetes				χ^2^ = 7.05	0.007
Yes	244 (16.44)	19.68	14.43		
No	1,240 (83.56)	80.32	85.57		
Geographic location of residence				χ^2^ = 92.73	<0.0001
Metropolitan	1,153 (77.70)	64.50	85.90		
Non-metropolitan	331 (22.30)	35.50	14.10		
Outcome	Mean ± SD ^b/^	Mean± SD ^b/^	Mean ± SD ^b/^		
Mini–Mental State Examination Score	27.23 ± 2.56	27.25 ± 2.39	27.21 ± 2.65	*t* = 0.31	0.759
	N (%)	%	%		
Decreased cognitive function^e/^	126 (8.49)	5.62	10.27	χ^2^ = 9.76	0.001

*There could be a round error in the percentages shown above*.

a/ *Rao-Scott Chi-Square test and t-test to compare the difference between males and females in terms of categorical variables and continuous variables, respectively*.

b/ *SD: standard deviation*.

c/ *Missing information were usually excluded except for smoking status, because most of women were likely to be non-smokers or social smokers*.

d/ *Created based on the Korean version of Short Geriatric Depression Scale (SGDS-K) (i.e., depressed = yes if 8 ≤ SGDS-K ≤ 15; depressed = no if 0 ≤ SGDS-K <8)*.

e/ *Decreased cognitive function was defined as MMSE score ≤ 23*.

Tables 2, 3 present results from our penalized logistic regression analyses to estimate the odds of decreased cognitive function associated with exposure to air pollutants (PM_10_, PM_2.5−10_, and NO_2_) by geographic location of residence and detect gender differences. Specifically, our interaction models (Table 2) find that women than men had a higher risk for decreased cognitive function associated with an increase in PM_10_ and PM_2.5−10_, respectively, even after adjusting for related factors [OR 1.01 [95%CI 1.00–1.03], *p* = 0.016 (PM10); OR 1.03 [95%CI 1.01–1.07], *p* = 0.023 (PM_2.5−10_)]. After stratification by metropolitan status, our results also find that the adverse effect of NO2 exposure on cognitive function was higher in women than men [OR 1.02 [95%CI 1.00–1.05], *p* = 0.024 (metropolitan); OR 1.12 [95%CI 1.04–1.20], *p* = 0.001 (non-metropolitan)]. Notably, the magnitude of the effect sizes was shown stronger among those in non-metropolitan regions than metropolitan ones, which indicates that the adverse effects of air pollution exposure were higher in non-metropolitan women compared with metropolitan women. Meanwhile, our stratified models (Table 3) find that among the metropolitan group, PM_2.5−10_ exposure was significantly associated with risk for decreased cognitive function in women (OR 1.21 [95%CI 1.02–1.44], *p* = 0.028), which, however, no significant result was found for men.

**Table 2:** The odds of declining cognitive function associated with exposure to air pollutants (PM_10_, PM_2.5−10_, NO_2_) by geographic location of residence and effect modification by gender.

	**All**	**Metropolitan**	**Non-metropolitan**
	**OR (95% CI)^a/^**	**OR (95% CI)^a/^**	**OR (95% CI)^a/^**
PM_10_^b/^	0.93 (0.84–1.04)	0.97 (0.85–1.10)	0.82 (0.65–1.02)
PM_2.5−10_^b/^	1.02 (0.91–1.15)	1.07 (0.93–1.24)	0.83 (0.64–1.06)
NO_2_^b/^	0.97 (0.92–1.01)	0.98 (0.93–1.04)	0.88 (0.78–0.99)
Female	2.38 (1.28–4.42)	2.21 (0.99–4.91)	5.45 (2.52–11.80)
(PM_10_ ^b/^)^*^(Female)	**1.01 (1.00–1.03)**	1.01 (0.99–1.03)	**1.04 (1.02–1.07)**
(PM_2.5−10_ ^b/^)^*^ (Female)	**1.03 (1.01–1.07)**	**1.04 (1.00–1.07)**	**1.13 (1.06–1.22)**
(NO_2_ ^b/^)^*^(Female)	1.01 (0.98–1.02)	**1.02 (1.00–1.05)**	**1.12 (1.04–1.20)**

a/ *OR: odds ratio; CI: confidence interval. The bold values mean statistically significant*.

*Analyses were conducted with adjustment for confounding factors (age, gender, marital status, alcohol drinking, systolic blood pressure, BMI, education, income, physical activity, quality of daily life, disease morbidities, being depressed, metropolitan status)*.

b/ *Air pollutants scale was increased by 10 units since 1 unit change was too small*.

**Table 3:** The odds of declining cognitive function associated with exposure to air pollutants (PM_10_, PM_2.5−10_, NO_2_) by gender and geographic location of residence.

	**Men**			**Women**		
	**All**	**Metropolitan**	**Non-metropolitan**	**All**	**Metropolitan**	**Non-metropolitan**
	**OR (95% CI)^a/^**	**OR (95% CI)^a/^**	**OR (95% CI)^a/^**	**OR (95% CI)^a/^**	**OR (95% CI)^a/^**	**OR (95% CI)^a/^**
PM_10_^b/^	1.01 (0.93–1.11)	0.77 (0.61–0.97)	1.10 (0.79–1.54)	1.04 (0.90–1.19)	1.09 (0.93–1.28)	0.83 (0.59–1.17)
PM_2.5−10_^b/^	1.04 (0.95–1.15)	0.89 (0.70–1.12)	1.12 (0.77–1.61)	1.12 (0.96–1.30)	**1.21 (1.02–1.44)**	0.88 (0.59–1.29)
NO_2_^b/^	1.01 (0.98–1.04)	0.92 (0.84–1.02)	1.02 (0.87–1.20)	1.00 (0.94–1.06)	1.02 (0.95–1.10)	0.90 (0.76–1.07)

a/ *OR, odds ratio; CI: confidence interval. The bold value means statistically significant*.

b/ *Air pollutants scale was increased by 10 units since 1 unit change was too small*.

Further, our generalized linear mixed models find that an increase in PM_2.5−10_ exposure had a negative effect on the MMSE score, even after adjusting for related factors (β = −0.11, *p* = 0.003). Similarly, our negative binomial regression model finds that an increase in PM_2.5−10_ exposure had an adverse, crude relationship with the MMSE score (β = −0.007, *p* = 0.021). The details about the results are provided in Table 4.

**Table 4:** The estimated effects of exposure to air pollutants (PM_10_, PM_2.5−10_, NO_2_) on MMSE score by geographic location of residence and effect modification.

	**Generalized linear mixed model^a/^**		**Negative binomial model**			
			**Metropolitan**		**Non-metropolitan**	
	**β^b/^**	***P*^b/^**	**β^b/^**	***P*^b/^**	**β^b/^**	***P*^b/^**
PM_10_	0.0140	0.670	−0.0004	0.903	0.0057	0.364
PM_2.5−10_	**−0.1106**	**0.003**	−0.0046	0.194	0.0054	0.440
NO_2_	0.0203	0.097	−0.0001	0.962	0.0027	0.367
Female	−0.3786	0.044	−0.0051	0.705	−0.0488	0.071
(PM_10_)^*^ (Female)	−0.0062	0.127	−0.0002	0.561	−0.0010	0.115
(PM_2.5−10_)^*^ (Female)	−0.0126	0.162	−0.0007	0.383	−0.0032	0.120
(NO_2_)^*^ (Female)	−0.0003	0.955	−0.0003	0.507	−0.0027	0.297

a/ *Incorporated geographic location of residence (metropolitan vs. non-metropolitan) as random effects*.

b/ *β, coefficient; P, p-value*.

*After adjustment for confounding factors (age, gender, marital status, alcohol drinking, systolic blood pressure, BMI, education, income, physical activity, quality of daily life, disease morbidities, being depressed, metropolitan status)*.

In sensitivity analyses, first, after using a different cut point (“24–25”), our stratified models find that among the metropolitan group, PM_2.5−10_ was associated with risk of lower cognition in women (OR 1.26 [95%CI 1.11–1.42], *p* = 0.0002), which, however, no significant result was found for men. After examining exposure to air pollutants (PM_10_, PM_2.5−10_, and NO_2_) as interval variables, there seemed to be a pattern showing a negative relationship between the higher quartile of outdoor air pollution and MMSE score in the metropolitan group, indicating that higher levels of air pollution exposure were associated with decreased cognitive function (for PM_10_, OR 1.18 [95%CI 0.36–3.83] (Q2 vs. Q1), OR 1.25 [95%CI 0.38–4.07] (Q3 vs. Q1); for PM_2.5−10_, OR 0.86 [95%CI 0.22–3.29] (Q3 vs. Q1), OR 0.94 [95%CI 0.25–3.59] (Q4 vs. Q1); for NO_2_, OR 0.54 [95%CI 0.09–3.35] (Q2 vs. Q1); OR 0.73 [95%CI 0.12–4.52] (Q3 vs. Q1). With the log-transformation of air pollutants, our results from the generalized linear mixed models find that PM_10_ and PM_2.5−10_ exposures, respectively, affected MMSE scores more adversely in women than men (β = −0.07, *p* = 0.041 (“${\text{PM}}_{10}^{*}$female”); β = −0.09, *p* = 0.05 (“${\text{PM}}_{2.5-10}^{*}$female”). Although our negative binomial models find no significant effect medication, our results show that among the metropolitan group, PM_2.5−10_ was negatively associated with the MMSE score (β = −0.161, *p* = 0.028), similar to the results from the models without the log-transformation. Furthermore, our zero-truncated negative binomial models for exploring a non-linear effect of air pollution exposure on cognition find that PM_2.5−10_ exposure had an adverse effect on cognitive function among the metropolitan group (β = −0.007, *p* = 0.006).

## Discussion

In this cross-sectional study of Korean older adults, we explored gender differences in the relation of cognitive functioning associated with particulate air pollution. Albeit not strong, we found that women than men had a higher risk for declining cognitive function associated with increased exposures to PM_10_ and PM_2.5−10_, and after stratification by geographic location of residence, the adverse effect of NO_2_ exposure on cognitive function was greater in women than men. Furthermore, our results from both negative binomial and generalized linear mixed models find that an increase in PM_2.5−10_ had an adverse effect on declining cognitive function. Our main findings are both congruent and incongruent with prior studies (11, 21, 22). For example, a study of 105 healthy children who were highly exposed to PM_2.5_ and ozone in Mexico City examined the role of other risk factors including Apolipoprotein 4, gender, and BMI on the risk of declining cognition and found that girls than boys may be more likely to develop cognitive deficits associated with air pollution exposure (22). In the meantime, Chen et al. (21) examined the impact of air pollution on individual cognitive performance using the data from the China Family Panel Studies and found a significant, adverse effect of air quality measured by the air pollution index on both the verbal and math test scores of the respondents. Especially, the adverse effects of air pollution on cognitive performance were higher in men compared with women. However, a study conducted with a sample from the National Survey of Health and Nutrition in Mexico did not find any significant modification by sex in the relationship between PM_2.5_ concentrations and cognitive function (43).

A probable explanation may apply for our finding that women than men may have a higher risk for declining cognition associated with increased exposures to PM_10_ and PM_2.5−10_. It may be due to the different neurological structure or system between men and women, which could have affected cognitive function associated with air pollution exposure differently in them. For example, prior research investigated the relationship between the structural brain organization and general intelligence in men and women and found that women than men have less gray matter and more white matter as related to intelligence (44). Moreover, Gallart-Palau et al. (45) examined the extent of gender-specific molecular differences in developing Alzheimer's disease with cerebrovascular disease and found that despite the presence of a similar amount of brain protein (i.e., myelin-associated glycoprotein) in men and women, women than men were more likely to receive a stronger influence from the pathology of white matter.

It is well-documented that there exist the effects of sex/gender in cognition-related research (46–50). For example, a literature review by Nebel and colleagues (46) explored how sex and gender played a role in Alzheimer's disease and attempted to discern the risk factors and found that women have a greater lifetime risk of developing Alzheimer's disease and burden of the disease impacts women more significantly than men. They also pointed out that women generally live longer than men and have a higher risk of having disease morbidities, including coronary heart disease, depression, and myocardial infarction, which are all risk factors for cognitive decline or impairment (46, 47). Furthermore, as a possible attribute of the sex effects in risk for cognitive decline, they suggest evidence that older women (aged 65 years or above) who received estrogen-containing hormone therapy had a doubled risk for dementia (48, 49), while those who began receiving hormone therapy early in the menopausal transition had a lower dementia risk, compared with those who did not. In addition to those risk factors, evidence indicates that the genetic component “APOE ε4” interacts with sex to affect the risk of cognitive decline. For instance, in a study of analyzing 5,400 ordinary people, women with APOE ε4 had a higher risk of dementia based on the Clinical Dementia Rating Score as compared to APOE ε4-negative women as well as men with APOE ε4 (50). Furthermore, other sex-/gender- differing factors (i.e., body size, stress, socioeconomic status, and gendered activities, including cleaning, cooking, etc.) could be potential attributes of the sex/gender difference in the relationship between air pollution exposure and cognitive function (51).

This study has some limitations. First, the 1,484 subjects of an elderly Korean cohort, including the 569 men and 915 women, could have increased by involving and encouraging more participation. Probably, a relatively small number of male subjects may explain why, overall, no significant results were found for men in our stratified models. Further, there could be other aspects attributed to limitations in our results, including small effect size related to a unit increase in air pollution exposure and the air pollutant prediction model with less resolution to estimate air pollution exposure precisely (52). Second, merely three air pollutants (PM_10_, PM_2.5−10_, and NO_2_) were explored in this study. However, to our knowledge, many more studies had already examined other pollutants, including PM_2.5_, ozone (O_3_), carbon monoxide (CO), etc. (12, 19, 21, 23, 53–55). Third, our findings may not be applied to particular outdoor air pollution, given the fact that air pollution, mainly as traffic-related, is a composite of particles and gases that could also be correlated one another (2). Fourth, there may be a possibility of social desirability bias concerning a high proportion of missing information about smoking status in women. It is likely because traditionally in Korea, people had considered female smoking more negatively compared to male smoking. Also, given the questionnaire for smoking status “whether a participant smoked more than five packs of cigarettes in each entire life,” it is possible that women than men could have been more reluctant to answer the question appropriately. Next, in spite of adjusting for related confounding factors indicated by the prior studies, there may be other factors that we did not control for. For example, we were unable to adjust for genetic component (i.e., APOE ε4) due to a lack of genetic information in our data. Also, there could be other related environmental factors, including noise, weather, etc. Nevertheless, in consideration of the number of subjects, it is believed that the study adjusted for associated factors to the greatest extent.

In spite of the limitations, the present study has several strengths. First, the study was conducted based on the four different regions in Korea, which allowed us to examine the effects of different air pollution exposure levels on cognitive function in elderly in Korea so that our findings may be applied to other elderly living in similar environments. Second, to our knowledge, this study was one of the first, based on a Korean older adult cohort, to explore the extent of gender difference in the relation of cognitive function associated with outdoor air pollution. Furthermore, this study used different regression models (i.e., penalized logistic regression, negative binomial regression, and generalized linear mixed models) and strategies, including subgroup analyses, as well as sensitivity analyses to investigate the extent of gender difference mentioned. Finally, our study adjusted not only for the known risk factors but also for other probable confounding factors, including geographic location, physical activity, and quality of daily life, which other studies less considered for adjustment.

In summary, we found the presence of gender differences in decreased cognitive function associated with increased exposure to outdoor air pollution in an elderly population without known neurological diseases. Despite some limitations in our results, the study findings suggest that the adverse effects of increased exposures to air pollutants such as PM_10_ and PM_2.5−10_ on cognition seemed to be higher among women than men, particularly among those in the metropolitan areas, the adverse effect of NO_2_ exposure on cognitive function appeared to be greater in women than men. Our study results were confirmed by the sensitivity analyses, which also suggest that women with higher levels of air pollution exposure than men were found to have lower MMSE scores. In spite of our findings, additional research is warranted to further explore gender differences in the effects of air pollution on cognition and the mechanism in consideration of a possibility of the potentially related factors and probable bias source. Furthermore, more critical, if the adverse effect of outdoor air pollution on cognitive function is collectively found to be stronger in women than men, developing and implementing prevention programs or interventions tailored on older women, particularly those with increased risk for air pollution exposure, should be necessarily considered.

## Data Availability Statement

The datasets for this manuscript are not publicly available because of the government policy that regulates the disclosure of the datasets concerning this study. Requests to access the datasets should be directed to CK (PREMAN@yuhs.ac).

## Ethics Statement

This study was carried out in accordance with the recommendations of the Institutional Review Board of the Yonsei University Health System with written informed consent from all subjects. All subjects gave written informed consent in accordance with the Declaration of Helsinki. The protocol was approved by the Institutional Review Board of the Yonsei University Health System Clinical Trial Centre (IRB Approval No. 4-2014-0359).

## Author Contributions

HK planned the study, performed statistical analyses, and prepared the manuscript, including the revision. JN, YN, S-BK, and SO involved in the collection of the cohort data. CK supervised the study, critically reviewed the manuscript, and contributed to revising the paper.

### Conflict of Interest

The authors declare that the research was conducted in the absence of any commercial or financial relationships that could be construed as a potential conflict of interest.

## References

[1] McGuire LC Ford ES Ajani UA. Cognitive functioning as a predictor of functional disability in later life. Am J Geriatr Psychiatry. (2006) 14:36–42. 10.1097/01.JGP.0000192502.10692.d6 16407580

[2] Power MC Weisskopf MG Alexeeff SE Coull BA Spiro A III Schwartz J. Traffic-related air pollution and cognitive function in a cohort of older men. Environ Health Perspect. (2010) 119:682–87. 10.1289/ehp.1002767 21172758 PMC3094421

[3] Kim KW Park JH Kim MH Kim MD Kim BJ Kim SK. A nationwide survey on the prevalence of dementia and mild cognitive impairment in South Korea. J Alzheimer's Dis. (2011) 23:281–91. 10.3233/JAD-2010-101221 21178286

[4] Anttila T Helkala EL Viitanen M Kåreholt I Fratiglioni L Winblad B. Alcohol drinking in middle age and subsequent risk of mild cognitive impairment and dementia in old age: a prospective population based study. BMJ. (2004) 329:539. 10.1136/bmj.38181.418958.BE 15304383 PMC516103

[5] Lopez OL Jagust WJ Dulberg C Becker JT DeKosky ST Fitzpatrick A. Risk factors for mild cognitive impairment in the cardiovascular health study cognition study: part 2. Arch Neurol. (2003) 60:1394–99. 10.1001/archneur.60.10.1394 14568809

[6] Meyer JS Rauch GM Rauch RA Haque A Crawford K. Cardiovascular and other risk factors for Alzheimer's disease and vascular dementia. Ann N Y Acad Sci. (2003) 903:411–23. 10.1111/j.1749-6632.2000.tb06393.x 10818532

[7] Meyer JS Rauch G Rauch RA Haque A. Risk factors for cerebral hypoperfusion, mild cognitive impairment, and dementia. Neurobiol Aging. (2000) 21:161–69. 10.1016/S0197-4580(00)00136-6 10867201

[8] Tervo S Kivipelto M Hänninen T Vanhanen M Hallikainen M Mannermaa A. Incidence and risk factors for mild cognitive impairment: a population-based three-year follow-up study of cognitively healthy elderly subjects. Dement Geriatr Cogn Disord. (2004) 17:196–203. 10.1159/000076356 14739544

[9] Hagger-Johnson G Sabia S Brunner EJ Shipley M Bobak M Marmot M. Combined impact of smoking and heavy alcohol use on cognitive decline in early old age: Whitehall II prospective cohort study. Br J Psychiatry. (2013) 203:120–25. 10.1192/bjp.bp.112.122960 23846998 PMC3730115

[10] Elias PK Elias MF D'Agostino RB Cupples LA Wilson PW Silbershatz H. NIDDM and blood pressure as risk factors for poor cognitive performance: the Framingham Study. Diabetes Care. (1997) 20:1388–95. 10.2337/diacare.20.9.1388 9283785

[11] Wu YC Lin YC Yu HL Chen JH Chen TF Sun Y. Association between air pollutants and dementia risk in the elderly. Alzheimer's Dementia. (2015) 1:220–8. 10.1016/j.dadm.2014.11.015 27239507 PMC4876896

[12] Oudin A Forsberg B Adolfsson AN Lind N Modig L Nordin M. Traffic-related air pollution and dementia incidence in northern Sweden: a longitudinal study. Environ Health Perspect. (2015) 124:306–12. 10.1289/ehp.1408322 26305859 PMC4786976

[13] Neuberger M Schimek MG Horak F Jr Moshammer H Kundi M Frischer T Acute effects of particulate matter on respiratory diseases, symptoms and functions:: epidemiological results of the Austrian Project on Health Effects of Particulate Matter (AUPHEP). Atmos Environ. (2004) 38:3971–81. 10.1016/j.atmosenv.2003.12.044

[14] Dominici F Peng RD Bell ML Pham L McDermott A Zeger SL. Fine particulate air pollution and hospital admission for cardiovascular and respiratory diseases. JAMA. (2006) 295:1127–34. 10.1001/jama.295.10.1127 16522832 PMC3543154

[15] Ki M. 2015 MERS outbreak in Korea: hospital-to-hospital transmission. Epidemiol Health. (2015) 37:e2015033. 10.4178/epih/e2015033 26212508 PMC4533026

[16] Baldacci S Maio S Cerrai S Sarno G Baïz N Simoni M. Allergy and asthma: Effects of the exposure to particulate matter and biological allergens. Respir Med. (2015) 109:1089–104. 10.1016/j.rmed.2015.05.017 26073963

[17] Du Y Xu X Chu M Guo Y Wang J. Air particulate matter and cardiovascular disease: the epidemiological, biomedical and clinical evidence. J Thorac Dis. (2016) 8:E8–19. 10.3978/j.issn.2072-1439.2015.11.37. 26904258 PMC4740122

[18] Gu X Liu Q Deng F Wang X Lin H Guo X Association between particulate matter air pollution and risk of depression and suicide: systematic review and meta-analysis. Br J Psychiatry. (2019) 5:1–2. 10.1192/bjp.2018.295 30719959

[19] Weuve J Puett RC Schwartz J Yanosky JD Laden F Grodstein F. Exposure to particulate air pollution and cognitive decline in older women. Arch Int Med. (2012) 172:219–27. 10.1001/archinternmed.2011.683 22332151 PMC3622279

[20] Ailshire JA Crimmins EM. Fine particulate matter air pollution and cognitive function among older US adults. Am J Epidemiol. (2014) 180:359–66. 10.1093/aje/kwu155 24966214 PMC4128773

[21] Chen X Zhang X Zhang X Smog in our brains: gender differences in the impact of exposure to air pollution on cognitive performance in China. Intl Food Policy Res Inst. (2017). IFPRI Discussion Paper 1619. Available online at: http://ebrary.ifpri.org/cdm/ref/collection/p15738coll2/id/131100

[22] Calderón-Garcidueñas L Jewells V Galaz-Montoya C van Zundert B Pérez-Calatayud A Ascencio-Ferrel E. Interactive and additive influences of Gender, BMI and Apolipoprotein 4 on cognition in children chronically exposed to high concentrations of PM2. 5 and ozone. APOE 4 females are at highest risk in Mexico City. Environ Res. (2016) 150: 411–22. 10.1016/j.envres.2016.06.026 27376929

[23] Carey IM Anderson HR Atkinson RW Beevers SD Cook DG Strachan DP. Are noise and air pollution related to the incidence of dementia? A cohort study in London, England. BMJ Open. (2018) 8:e022404. 10.1136/bmjopen-2018-022404 30206085 PMC6144407

[24] Kim HC Oh SM. Noncommunicable diseases: current status of major modifiable risk factors in Korea. J Prev Med Public Health. (2013) 46:165. 10.3961/jpmph.2013.46.4.165 23946874 PMC3740221

[25] KCDCP. Survey Questionnaire and Investigation Tool. Available online at: https://cdc.go.kr/menu.es?mid=a40504100100 (accessed October 25, 2019).

[26] Shulman KI Herrmann N Brodaty H. IPA survey of brief cognitive screening instruments. Int Psychogeriatr. (2006) 18:281–94. 10.1017/S1041610205002693 16466586

[27] Mitchell AJ. A meta-analysis of the accuracy of the mini-mental state examination in the detection of dementia and mild cognitive impairment. J Psychiatry Res. (2009) 43:411–31. 10.1016/j.jpsychires.2008.04.014 18579155

[28] Grober E Hall C Mcginn M Nicholls T Stanford S Ehrlich A. Neuropsychological strategies for detecting early dementia. J Int Neuropsychol Soc. (2008) 14:130–42. 10.1017/S1355617708080156 18078539 PMC2763484

[29] Folstein MF Folstein SE McHugh PR. “Mini-mental state”: a practical method for grading the cognitive state of patients for the clinician. J Psychiatry Res. (1975) 12:189–98. 10.1016/0022-3956(75)90026-6 1202204

[30] Nilsson LG Bäckman L Erngrund K Nyberg L Adolfsson L Bucht G The Betula prospective cohort study: memory, health, and aging. Neuropsychol Dev Cogn B Aging Neuropsychol Cogn. (1997) 4:1–32. 10.1080/13825589708256633

[31] Kim SY Song I. National-scale exposure prediction for long-term concentrations of particulate matter and nitrogen dioxide in South Korea. Environ Pollut. (2017) 226:21–9. 10.1016/j.envpol.2017.03.056 28399503

[32] Launer LJ Andersen K Dewey M Letenneur L Ott A Amaducci LA. Rates and risk factors for dementia and Alzheimer's disease: results from EURODEM pooled analyses. Neurology. (1999) 52:78–84. 10.1212/WNL.52.1.78 9921852

[33] Cheng G Huang C Deng H Wang H. Diabetes as a risk factor for dementia and mild cognitive impairment: a meta-analysis of longitudinal studies. Intern Med J. (2012) 42:84–91. 10.1111/j.1445-5994.2012.02758.x 22372522

[34] Sharp SI Aarsland D Day S Sønnesyn H Alzheimer's Society Vascular Dementia Systematic Review Group Ballard C. Hypertension is a potential risk factor for vascular dementia: systematic review. Int J Geriatr Psychiatry. (2011) 26:661–9. 10.1002/gps.2572 21495075

[35] Lim JU Lee JH Kim JS Hwang YI Kim TH Lim SY. Comparison of World Health Organization and Asia-Pacific body mass index classifications in COPD patients. Int J Chron Obstruct Pulmon Dis. (2017) 12:2465–75. 10.2147/COPD.S141295 28860741 PMC5571887

[36] WHO/IASO/IOTF The Asia-Pacific Perspective: Redefining Obesity and Its Treatment. Health Communications Australia: Melbourne (2000).

[37] Bae JN Cho MJ. Development of the Korean version of the Geriatric Depression Scale and its short form among elderly psychiatric patients. J Psychosom Res. (2004) 57:297–305. 10.1016/j.jpsychores.2004.01.004 15507257

[38] Laurin D Verreault R Lindsay J MacPherson K Rockwood K. Physical activity and risk of cognitive impairment and dementia in elderly persons. Arch Neurol. (2001) 58:498–504. 10.1001/archneur.58.3.498 11255456

[39] Moyle W O'Dwyer S. Quality of life in people living with dementia in nursing homes. Curr Opin Psychiatr. (2012) 25:480–84. 10.1097/YCO.0b013e32835a1ccf 23037963

[40] Wang Z Ma S Wang CY. Variable selection for zero-inflated and overdispersed data with application to health care demand in Germany. Biom J. (2015) 57:867–84. 10.1002/bimj.201400143 26059498 PMC5525141

[41] Eilers PH Boer JM van Ommen GJ van Houwelingen HC Classification of microarray data with penalized logistic regression. In: Microarrays: Optical technologies and informatics. International Society for Optics and Photonics. San Jose, CA (2001). p. 187–99. 10.1117/12.427987

[42] Feng C Wang H Lu N Chen T He H Lu Y. Log-transformation and its implications for data analysis. Shanghai Arch. Psychiatry. (2014) 26:105–9. 10.3969/j.issn.1002-0829.2014.02.009 25092958 PMC4120293

[43] Salinas-Rodríguez A Fernández-Niño JA Manrique-Espinoza B Moreno-Banda GL Sosa-Ortiz AL Qian ZM. Exposure to ambient PM2. 5 concentrations and cognitive function among older Mexican adults. Environ Int. (2018) 117:1–9. 10.1016/j.envint.2018.04.033 29704751

[44] Haier RJ Jung RE Yeo RA Head K Alkire M T. The neuroanatomy of general intelligence: sex matters. NeuroImage. (2005) 25:320–27. 10.1016/j.neuroimage.2004.11.019 15734366

[45] Gallart-Palau X Lee BS Adav SS Qian J Serra A Park JE. Gender differences in white matter pathology and mitochondrial dysfunction in Alzheimer's disease with cerebrovascular disease. Mol Brain. (2016) 9:27. 10.1186/s13041-016-0205-7 26983404 PMC4794845

[46] Nebel RA Aggarwal NT Barnes LL Gallagher A Goldstein JM Kantarci K. Understanding the impact of sex and gender in Alzheimer's disease: a call to action. Alzheimers Dement. (2018) 14:1171–83. 10.1016/j.jalz.2018.04.008 29907423 PMC6400070

[47] Kautzky-Willer A Harreiter J Pacini G. Sex and gender differences in risk, pathophysiology and complications of type 2 diabetes mellitus. Endocr Rev. (2016) 37:278–316. 10.1210/er.2015-1137 27159875 PMC4890267

[48] Shumaker SA Legault C Rapp SR Thal L Wallace RB Ockene JK. Estrogen plus progestin and the incidence of dementia and mild cognitive impairment in postmenopausal women: the Women's Health Initiative Memory Study: a randomized controlled trial. JAMA. (2003) 289:2651–62. 10.1001/jama.289.20.2651 12771112

[49] Shumaker S Legault C Kuller L Rapp SR Thal L Lane DS. Conjugated equine estrogens and incidence of probable dementia and mild cognitive impairment in postmenopausal women: Women's Health Initiative Memory Study. JAMA. (2004) 291:2947–58. 10.1001/jama.291.24.2947 15213206

[50] Altmann A Tian L Henderson VW Greicius MD Alzheimer's Disease Neuroimaging Initiative Investigators. Sex modifies the APOE-related risk of developing Alzheimer disease. Ann Neurol. (2014) 75:563–73. 10.1002/ana.24135 24623176 PMC4117990

[51] Clougherty JE. A growing role for gender analysis in air pollution epidemiology. Environ Health Perspect. (2009) 118:167–76. 10.1289/ehp.0900994 20123621 PMC2831913

[52] Schmidt S. Particulate matter and cognition: using brain imaging to study impacts of air pollution. Environ Health Perspect. (2018) 126:064003. 10.1289/EHP3445 29883072 PMC6108578

[53] Gatto NM Henderson VW Hodis HN St John JA Lurmann F Chen JC. Components of air pollution and cognitive function in middle-aged and older adults in Los Angeles. Neurotoxicology. (2014) 40:1–7. 10.1016/j.neuro.2013.09.004 24148924 PMC3946571

[54] Pages B Planton M Buys S Lemesle B Birmes P Barbeau EJ. Neuropsychological outcome after carbon monoxide exposure following a storm: a case-control study. BMC Neurol. (2014) 14:153. 10.1186/1471-2377-14-153. 25048040 PMC4118199

[55] Chen JC Schwartz J. Neurobehavioral effects of ambient air pollution on cognitive performance in US adults. Neurotoxicology. (2009) 30:231–9. 10.1016/j.neuro.2008.12.011 19150462

